# Paralysie faciale zostérienne

**DOI:** 10.11604/pamj.2015.22.238.8105

**Published:** 2015-11-13

**Authors:** Rim Lahiani, Madiha Mahfoudhi

**Affiliations:** 1 Service ORL, Hôpital Charles Nicolle, Tunis, Tunisie; 2 Service de Médecine Interne A, Hôpital Charles Nicolle, Tunis, Tunisie

**Keywords:** Paralysie faciale périphérique, zona, antiviral, peripheral facial paralysis, shingles, antiviral

## Image en medicine

L'origine zostérienne représente 3 à 12% des paralysies faciales périphériques. Elle survient en général une seule fois dans la vie chez les sujets ayant eu une primo-infection à virus varicelle-zona. Le diagnostic virologique et sérologique prend tout son intérêt dans les formes graves et atypiques. Une étiologie zostérienne doit être évoquée si une paralysie faciale douloureuse est accompagnée d'une éruption et de troubles audio-vestibulaires. Le pronostic est péjoratif malgré un traitement précoce. Une décompression chirurgicale n'est envisagée qu'après une analyse pronostique raisonnée. Patiente âgée de 63 ans, hypertendue, aux antécédents de varicelle, a consulté pour asymétrie faciale, otodynie et lésions cutanées de la face et l'oreille gauche. Elle se plaignait d'une hypoesthésie de la face ainsi que des douleurs pharyngées évoluant depuis 5 jours. L'examen physique a objectivé des vésicules cutanées correspondant à des lésions de zona de la zone de Ramsay Hunt et de l'hémiface gauche associées à une paralysie faciale périphérique gauche. L'examen biologique a retrouvé un syndrome inflammatoire. L'IRM a révélé une névrite du nerf facial gauche. Le traitement s'est basé sur un antiviral (Aciclovir) pendant 10 jours, un antalgique, des soins locaux et une kinésithérapie motrice. L’évolution était marquée par une régression des lésions vésiculeuses et une amélioration partielle de la paralysie faciale avec passage au grade II en 2 mois.

**Figure 1:**
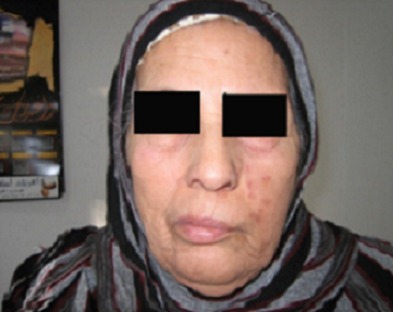
Paralysie faciale périphérique gauche

